# The genome sequence of the hawkweed Cheilosia, *Cheilosia urbana *(Meigen, 1822)

**DOI:** 10.12688/wellcomeopenres.19569.2

**Published:** 2024-10-29

**Authors:** Steven Falk, Iva Gorše, University of Oxford and Wytham Woods Genome Acquisition Lab, Darwin Tree of Life Barcoding collective, Wellcome Sanger Institute Tree of Life programme, Wellcome Sanger Institute Scientific Operations: DNA Pipelines collective, Tree of Life Core Informatics collective, Darwin Tree of Life Consortium

**Affiliations:** 1 Independent researcher, Kenilworth, England, UK; 2 Department of Biology and Ecology, University of Novi Sad, Novi Sad, Serbia

**Keywords:** Cheilosia urbana, hawkweed Cheilosia, genome sequence, chromosomal, Diptera

## Abstract

We present a genome assembly from an individual female *Cheilosia urbana* (the hawkweed Cheilosia; Arthropoda; Insecta; Diptera; Syrphidae). The genome sequence is 546.9 megabases in span. Most of the assembly is scaffolded into 5 chromosomal pseudomolecules, including the X sex chromosome. The mitochondrial genome has also been assembled and is 17.08 kilobases in length.

## Species taxonomy

Eukaryota; Metazoa; Eumetazoa; Bilateria; Protostomia; Ecdysozoa; Panarthropoda; Arthropoda; Mandibulata; Pancrustacea; Hexapoda; Insecta; Dicondylia; Pterygota; Neoptera; Endopterygota; Diptera; Brachycera; Muscomorpha; Eremoneura; Cyclorrhapha; Aschiza; Syrphoidea; Syrphidae; Eristalinae; Rhingiini; *Cheilosia*; *Cheilosia urbana* ( Meigen, 1822) (NCBI:txid173985).

## Background

*Cheilosia urbana* ( Meigen, 1822) is a common and widespread Palaearctic spring-flying species, that inhabits grasslands as well as open areas in both coniferous and deciduous forests and scrublands ( Speight, 2020). This hoverfly occurs from the United Kingdom eastward through central and southern Europe to the Balkans and Turkey. The species is listed as “Least Concern” on the IUCN Red List ( Vujić & Šebić, 2021).

The flight season extends from April to June, and even to July at higher altitudes and/or more northern latitudes. Adults have been found to visit a variety of flowers, including *Acer pseudoplatanus* L, *Anemone nemorosa* L., *Prunus spinosa* L. and species of the genera *Salix*, *Taraxacum*, *Euphorbia* and *Potentilla*.

In addition to pollination service, individuals of *C. urbana* also have a role as biological control agents. The English common name ‘hawkweed Cheilosia’ derives from its association with mouse-ear hawkweed, *Hieracium pilosella* L. ( Doczkal, 1996). The females oviposit on leaf axils, afterwards larvae migrate into the soil and feed externally on the roots of the plant, in which they make small holes. Moreover, Grosskopf *et al*. (2002) revealed that neonate *C. urbana* larvae fed and completed development to the adult stage on eight different *Hieracium* spp., approving the potential of this hoverfly in management strategy of weeds. This is of particular importance in New Zealand, as well as the North and South Americas, where *Hieracium* species of Eurasian origin represent invasive alien plants that threaten to cause great economic and environmental harm ( Grosskopf, 2005; Grosskopf *et al.*, 2002).

This is the first production of a high-quality *C. urbana* genome and we believe that the sequence described here, generated as part of the Darwin Tree of Life project, will further aid understanding of the biology and ecology of this hoverfly. These data can also help in resolving taxonomic issues present in certain species groups of *Cheilosia* and other hoverfly genera.

## Genome sequence report

**Figure 1.**
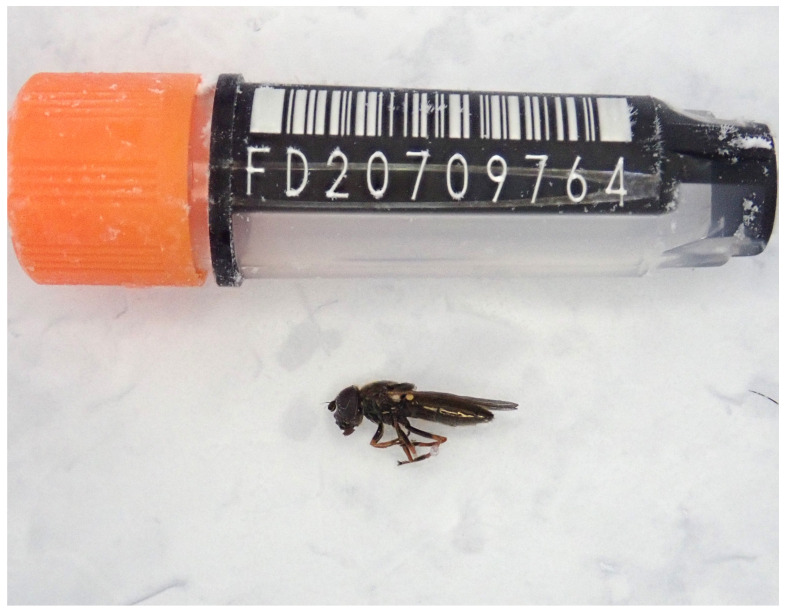
Photograph of the *Cheilosia urbana* (idCheUrba1) specimen used for genome sequencing.

The genome was sequenced from one female *Cheilosia urbana* ( Figure 1) collected from Wytham Woods, Oxfordshire, UK (51.76, –1.34). A total of 32-fold coverage in Pacific Biosciences single-molecule HiFi long reads was generated. Primary assembly contigs were scaffolded with chromosome conformation Hi-C data. Manual assembly curation corrected 48 missing joins or mis-joins and removed 6 haplotypic duplications, reducing the assembly length by 0.51% and the scaffold number by 41.94%, and increasing the scaffold N50 by 1.65%.

**Figure 2.**
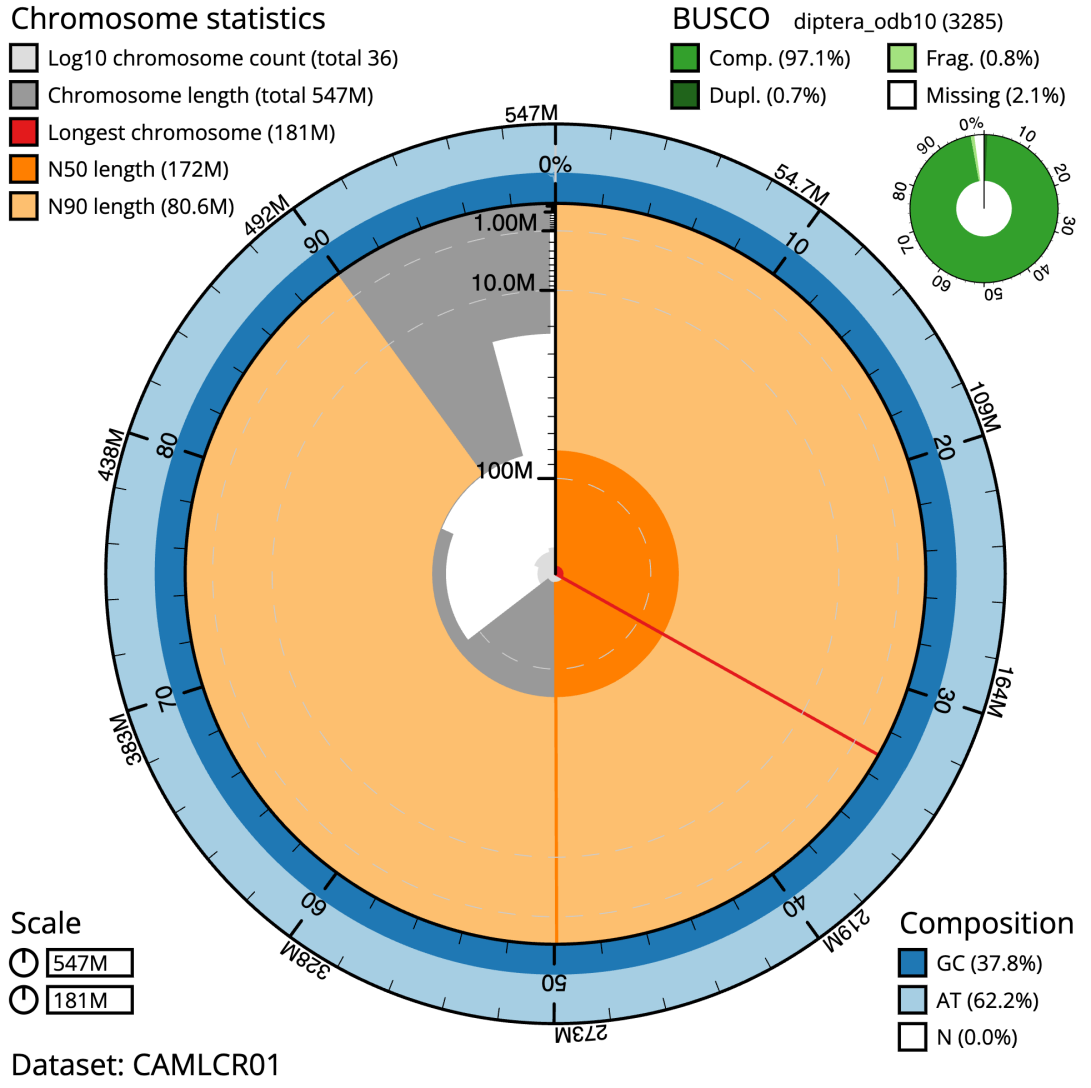
Genome assembly of *Cheilosia urbana*, idCheUrba1.1: metrics. The BlobToolKit Snailplot shows N50 metrics and BUSCO gene completeness. The main plot is divided into 1,000 size-ordered bins around the circumference with each bin representing 0.1% of the 546,942,332 bp assembly. The distribution of scaffold lengths is shown in dark grey with the plot radius scaled to the longest scaffold present in the assembly (181,276,847 bp, shown in red). Orange and pale-orange arcs show the N50 and N90 scaffold lengths (171,959,956 and 80,646,311 bp), respectively. The pale grey spiral shows the cumulative scaffold count on a log scale with white scale lines showing successive orders of magnitude. The blue and pale-blue area around the outside of the plot shows the distribution of GC, AT and N percentages in the same bins as the inner plot. A summary of complete, fragmented, duplicated and missing BUSCO genes in the diptera_odb10 set is shown in the top right. An interactive version of this figure is available at https://blobtoolkit.genomehubs.org/view/idCheUrba1.1/dataset/CAMLCR01/snails.

The final assembly has a total length of 546.9 Mb in 35 sequence scaffolds with a scaffold N50 of 172.0 Mb ( Table 1). Most (99.7%) of the assembly sequence was assigned to 5 chromosomal-level scaffolds, representing 4 autosomes and the X sex chromosome. The assemblies used as comparators to identify the X chromosome were *Cheilosia pagana* (GCA_936431705.1) and *Cheilosia vulpina* (GCA_916610125).1. Chromosome-scale scaffolds confirmed by the Hi-C data are named in order of size ( Figure 2– Figure 5; Table 2). While not fully phased, the assembly deposited is of one haplotype. Contigs corresponding to the second haplotype have also been deposited. The mitochondrial genome was also assembled and can be found as a contig within the multifasta file of the genome submission.

**Table 1.** Genome data for *Cheilosia urbana*, idCheUrba1.1.

Project accession data		
Assembly identifier	idCheUrba1.1	
Species	*Cheilosia urbana*	
Specimen	idCheUrba1	
NCBI taxonomy ID	173985	
BioProject	PRJEB54805	
BioSample ID	SAMEA10166768	
Isolate information	idCheUrba1, female: whole organism (DNA sequencing) idCheUrba2, male: whole organism (Hi-C scaffolding)	
Assembly metrics *		*Benchmark*
Consensus quality (QV)	65.1	*≥ 50*
*k*-mer completeness	100%	*≥ 95%*
BUSCO **	C:97.1%[S:96.4%,D:0.7%], F:0.8%,M:2.1%,n:3,285	*C ≥ 95%*
Percentage of assembly mapped to chromosomes	99.7%	*≥ 95%*
Sex chromosomes	X chromosome	*localised homologous pairs*
Organelles	Mitochondrial genome assembled	*complete single alleles*
Raw data accessions		
PacificBiosciences SEQUEL II	ERR9981095	
Hi-C Illumina	ERR9988137	
Genome assembly		
Assembly accession	GCA_946477585.1	
*Accession of alternate haplotype*	GCA_946477595.1	
Span (Mb)	546.9	
Number of contigs	152	
Contig N50 length (Mb)	11.8	
Number of scaffolds	36	
Scaffold N50 length (Mb)	172.0	
Longest scaffold (Mb)	181.3	

* Assembly metric benchmarks are adapted from column VGP-2020 of “Table 1: Proposed standards and metrics for defining genome assembly quality” from ( Rhie *et al.*, 2021). ** BUSCO scores based on the diptera_odb10 BUSCO set using v5.3.2. C = complete [S = single copy, D = duplicated], F = fragmented, M = missing, n = number of orthologues in comparison. A full set of BUSCO scores is available at https://blobtoolkit.genomehubs.org/view/idCheUrba1.1/dataset/CAMLCR01/busco.

**Figure 3.**
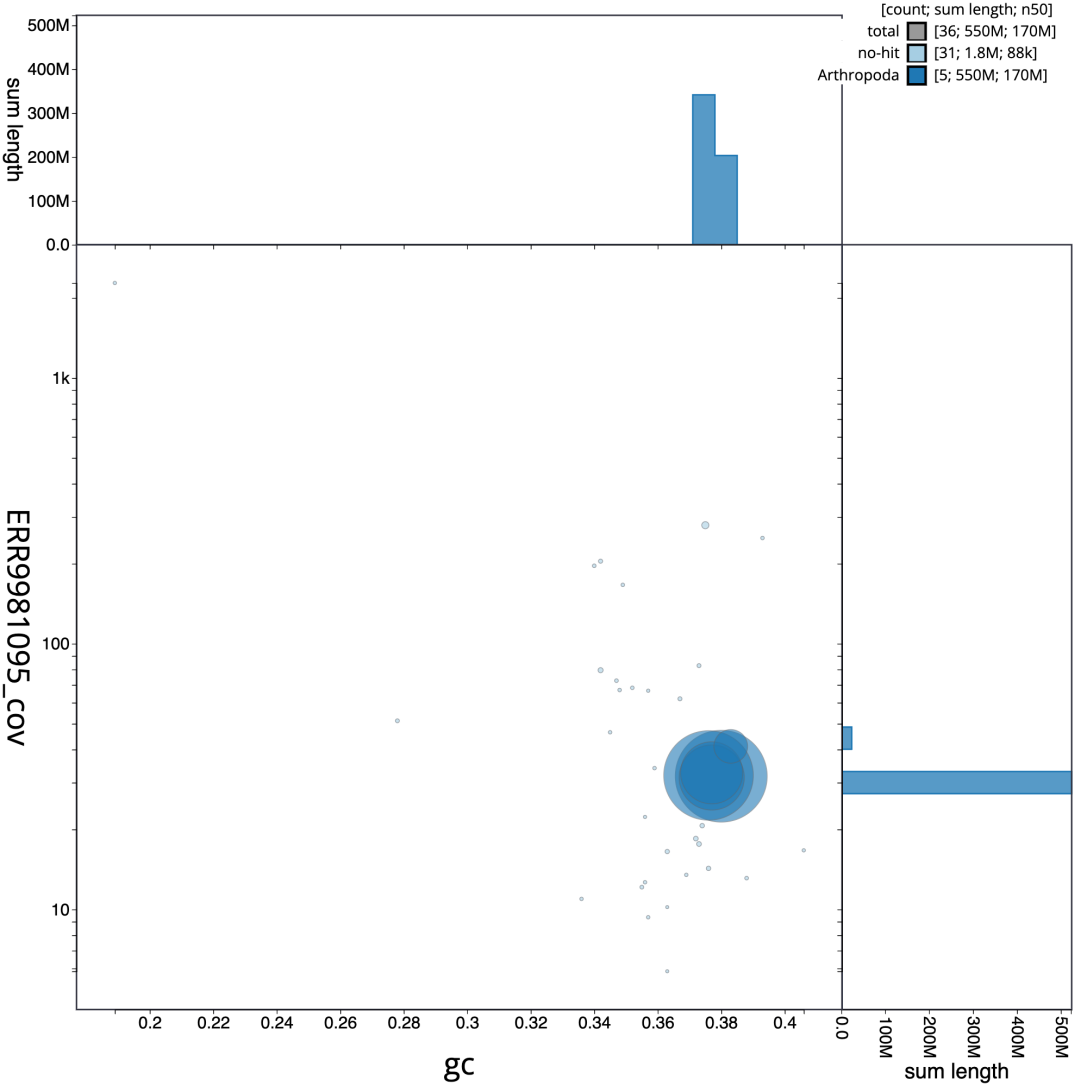
Genome assembly of *Cheilosia urbana*, idCheUrba1.1: BlobToolKit GC-coverage plot. Scaffolds are coloured by phylum. Circles are sized in proportion to scaffold length. Histograms show the distribution of scaffold length sum along each axis. An interactive version of this figure is available at https://blobtoolkit.genomehubs.org/view/idCheUrba1.1/dataset/CAMLCR01/blob.

**Figure 4.**
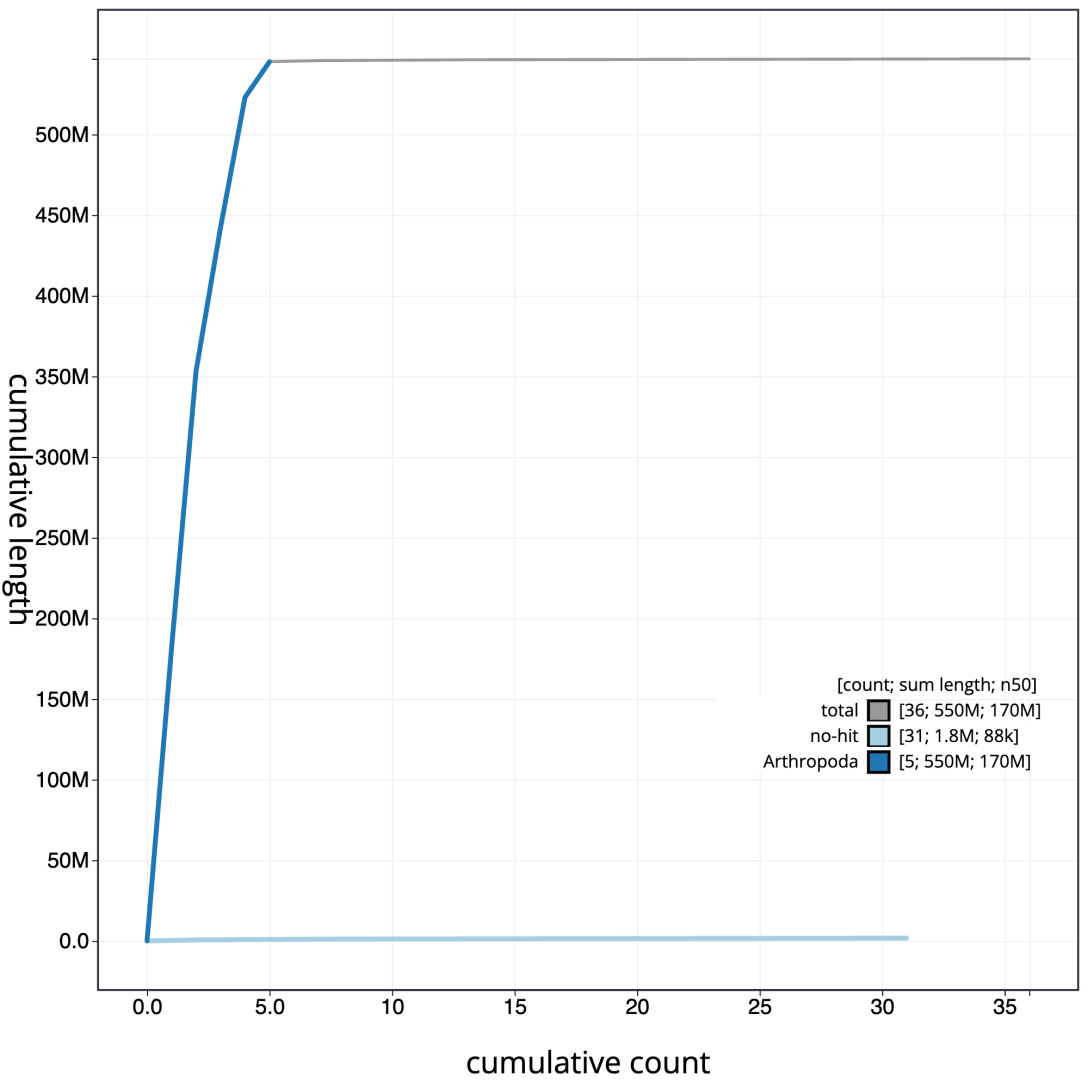
Genome assembly of *Cheilosia urbana*, idCheUrba1.1: BlobToolKit cumulative sequence plot. The grey line shows cumulative length for all scaffolds. Coloured lines show cumulative lengths of scaffolds assigned to each phylum using the buscogenes taxrule. An interactive version of this figure is available at https://blobtoolkit.genomehubs.org/view/idCheUrba1.1/dataset/CAMLCR01/cumulative.

**Figure 5.**
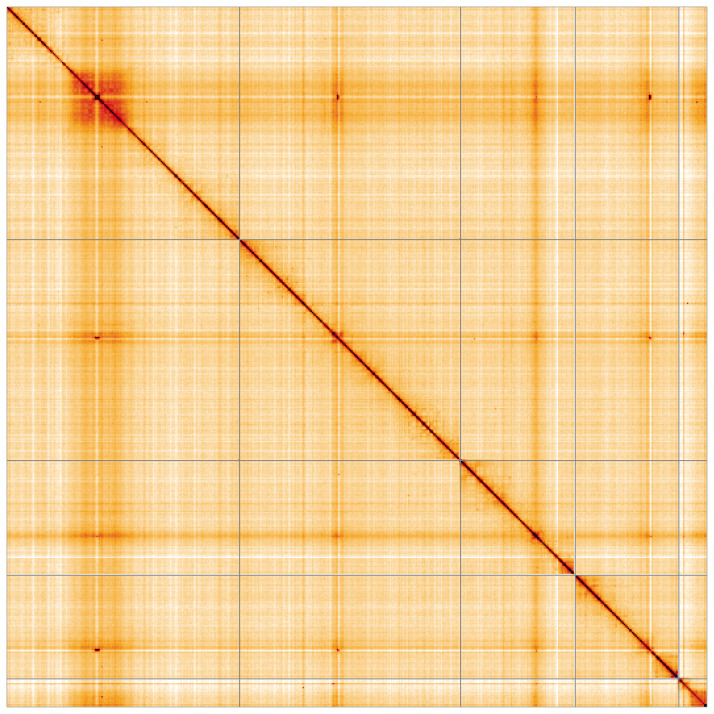
Genome assembly of *Cheilosia urbana*, idCheUrba1.1: Hi-C contact map of the idCheUrba1.1 assembly, visualised using HiGlass. Chromosomes are shown in order of size from left to right and top to bottom. An interactive version of this figure may be viewed at https://genome-note-higlass.tol.sanger.ac.uk/l/?d=LxrU8VG5TYuxrLOcPeXiKg.

**Table 2.** Chromosomal pseudomolecules in the genome assembly of *Cheilosia urbana*, idCheUrba1.

INSDC accession	Chromosome	Length (Mb)	GC%
OX297853.1	1	181.28	38.0
OX297854.1	2	171.96	37.5
OX297855.1	3	89.2	37.5
OX297856.1	4	80.65	37.5
OX297857.1	X	22.08	38.5
OX297858.1	MT	0.02	18.5

The estimated Quality Value (QV) of the final assembly is 65.1 with *k*-mer completeness of 100%, and the assembly has a BUSCO v5.3.2 completeness of 97.1% (single = 96.4%, duplicated = 0.7%), using the diptera_odb10 reference set ( *n* = 3,285).

Metadata for specimens, spectral estimates, sequencing runs, contaminants and pre-curation assembly statistics can be found at https://links.tol.sanger.ac.uk/species/173985.

## Methods

### Sample acquisition and nucleic acid extraction

The specimen selected for genome sequencing was a female *Cheilosia urbana* (specimen ID Ox001288, individual idCheUrba1, while the specimen used for Hi-C scaffolding was a male *C. urbana* (specimen ID Ox001310, individual idCheUrba2). Both specimens were netted in Wytham Woods, Oxfordshire (biological vice-county Berkshire), UK (latitude 51.76, longitude –1.34) on 2021-04-23. Steven Falk (independent researcher) collected and identified the specimens. The specimens were snap-frozen on dry ice.

The specimen was prepared for DNA extraction at the Tree of Life laboratory, Wellcome Sanger Institute (WSI). The idCheUrba1 sample was weighed and dissected on dry ice. Whole organism tissue was disrupted using a Nippi Powermasher fitted with a BioMasher pestle. DNA was extracted at the Wellcome Sanger Institute (WSI) Scientific Operations core using the Qiagen MagAttract HMW DNA kit, according to the manufacturer’s instructions.

### Sequencing

Pacific Biosciences HiFi circular consensus DNA sequencing libraries were constructed according to the manufacturers’ instructions. DNA sequencing was performed by the Scientific Operations core at the WSI on a Pacific Biosciences SEQUEL II (HiFi) instrument. Hi-C data were also generated from whole organism tissue of idCheUrba2 using the Arima2 kit and sequenced on the Illumina NovaSeq 6000 instrument.

### Genome assembly, curation and evaluation

Assembly was carried out with Hifiasm ( Cheng *et al.*, 2021) and haplotypic duplication was identified and removed with purge_dups ( Guan *et al.*, 2020). The assembly was then scaffolded with Hi-C data ( Rao *et al.*, 2014) using YaHS ( Zhou *et al.*, 2023). The assembly was checked for contamination and corrected as described previously ( Howe *et al.*, 2021). Manual curation was performed using HiGlass ( Kerpedjiev *et al.*, 2018) and Pretext ( Harry, 2022). The mitochondrial genome was assembled using MitoHiFi ( Uliano-Silva *et al.*, 2022), which runs MitoFinder ( Allio *et al.*, 2020) or MITOS ( Bernt *et al.*, 2013) and uses these annotations to select the final mitochondrial contig and to ensure the general quality of the sequence.

### Evaluation of genome assembly

The final assembly was post-processed and evaluated with the three Nextflow ( Di Tommaso *et al.*, 2017) DSL2 pipelines “sanger-tol/readmapping” ( Surana *et al.*, 2023a), “sanger-tol/genomenote” ( Surana *et al.*, 2023b). The pipeline sanger-tol/readmapping aligns the Hi-C reads with bwa-mem2 ( Vasimuddin *et al.*, 2019) and combines the alignment files with SAMtools ( Danecek *et al.*, 2021). The sanger-tol/genomenote pipeline transforms the Hi-C alignments into a contact map with BEDTools ( Quinlan & Hall, 2010) and the Cooler tool suite ( Abdennur & Mirny, 2020), which is then visualised with HiGlass ( Kerpedjiev *et al.*, 2018). It also provides statistics about the assembly with the NCBI datasets report ( Sayers *et al.*, 2024). For the *k*-mer completeness and QV consensus quality values, the *k*-mer database is made of the PacBio reads from ERR9981095 and *k*=31. Reads containing adapter sequences have been filtered out. computes k-mer completeness and QV consensus quality values with FastK and MERQURY.FK ( Rhie *et al.*, 2020), and a completeness assessment with BUSCO ( Manni *et al.*, 2021). The genome was also analysed within the BlobToolKit environment ( Challis *et al.*, 2020).

Table 3 contains a list of relevant software tool versions and sources.

**Table 3.** Software tools: versions and sources.

Software tool	Version	Source
BEDTools	2.30.0	https://github.com/arq5x/bedtools2
BlobToolKit	4.1.5	https://github.com/blobtoolkit/blobtoolkit
BUSCO	5.3.2	https://gitlab.com/ezlab/busco
bwa-mem2	2.2.1	https://github.com/bwa-mem2/bwa-mem2
Cooler	0.8.11	https://github.com/open2c/cooler
FastK	427104ea91c78c3b8b8b49f1a7d6bbeaa869ba1c	https://github.com/thegenemyers/FASTK
Hifiasm	0.16.1-r375	https://github.com/chhylp123/hifiasm
HiGlass	1.11.6	https://github.com/higlass/higlass
Merqury	MerquryFK	https://github.com/thegenemyers/MERQURY.FK
MitoHiFi	2	https://github.com/marcelauliano/MitoHiFi
NCBI Datasets	15.12.0	https://github.com/ncbi/datasets
PretextView	0.2	https://github.com/wtsi-hpag/PretextView
purge_dups	1.2.3	https://github.com/dfguan/purge_dups
samtools	1.16.1, 1.17, and 1.18	https://github.com/samtools/samtools
sanger-tol/genomenote	v1.0	https://github.com/sanger-tol/genomenote
sanger-tol/readmapping	1.1.0	https://github.com/sanger-tol/readmapping/ tree/1.1.0
YaHS	yahs-1.1.91eebc2	https://github.com/c-zhou/yahs

### Wellcome Sanger Institute – Legal and Governance

The materials that have contributed to this genome note have been supplied by a Darwin Tree of Life Partner. The submission of materials by a Darwin Tree of Life Partner is subject to the **‘Darwin Tree of Life Project Sampling Code of Practice’**, which can be found in full on the Darwin Tree of Life website here. By agreeing with and signing up to the Sampling Code of Practice, the Darwin Tree of Life Partner agrees they will meet the legal and ethical requirements and standards set out within this document in respect of all samples acquired for, and supplied to, the Darwin Tree of Life Project.

Further, the Wellcome Sanger Institute employs a process whereby due diligence is carried out proportionate to the nature of the materials themselves, and the circumstances under which they have been/are to be collected and provided for use. The purpose of this is to address and mitigate any potential legal and/or ethical implications of receipt and use of the materials as part of the research project, and to ensure that in doing so we align with best practice wherever possible. The overarching areas of consideration are:

- Ethical review of provenance and sourcing of the material
- Legality of collection, transfer and use (national and international)

Each transfer of samples is further undertaken according to a Research Collaboration Agreement or Material Transfer Agreement entered into by the Darwin Tree of Life Partner, Genome Research Limited (operating as the Wellcome Sanger Institute), and in some circumstances other Darwin Tree of Life collaborators.

## Data Availability

European Nucleotide Archive: *Cheilosia urbana* (hawkweed Cheilosia). Accession number PRJEB54805; https://identifiers.org/ena.embl/PRJEB54805. ( Wellcome Sanger Institute, 2022) The genome sequence is released openly for reuse. The *Cheilosia urbana* genome sequencing initiative is part of the Darwin Tree of Life (DToL) project. All raw sequence data and the assembly have been deposited in INSDC databases. The genome will be annotated using available RNA-Seq data and presented through the Ensembl pipeline at the European Bioinformatics Institute. Raw data and assembly accession identifiers are reported in Table 1.

## Author information

Members of the University of Oxford and Wytham Woods Genome Acquisition Lab are listed here: https://doi.org/10.5281/zenodo.4789928.

Members of the Darwin Tree of Life Barcoding collective are listed here: https://doi.org/10.5281/zenodo.4893703.

Members of the Wellcome Sanger Institute Tree of Life programme are listed here: https://doi.org/10.5281/zenodo.4783585.

Members of Wellcome Sanger Institute Scientific Operations: DNA Pipelines collective are listed here: https://doi.org/10.5281/zenodo.4790455.

Members of the Tree of Life Core Informatics collective are listed here: https://doi.org/10.5281/zenodo.5013541.

Members of the Darwin Tree of Life Consortium are listed here: https://doi.org/10.5281/zenodo.4783558.
