# Supplementary material for: The genome sequence of the hawkweed Cheilosia, Cheilosia urbana (Meigen, 1822)
Source: Wellcome Open Res. 2024 Oct 29;8:311. Originally published 2023 Jul 19. [Version 2] doi: 10.12688/wellcomeopenres.19569.2 (PMC11282393; doi:10.12688/wellcomeopenres.19569.2)
Supplement: Peer review history

# Reviewer report: Reviewer response for version 2

**DOI:** 10.21956/wellcomeopenres.25256.r108773

**Published:** 2024-11-11

**Authors:** Thomas Brown

**Affiliations:** 1 Leibniz IZW (Berlin) & ERGA/BGP, Leibniz, Germany

Thank you for the second submission of this article. i appreciate the expanded introduction to give some more context of the use of this particular genome in the context of this species.

 Thank you as well for explicitly stating the use of FastK and Merqury.FK in the methods section. I am still surprised about the values obtained, but understand where they have come from now.

Are sufficient details of methods and materials provided to allow replication by others?

Partly

Is the rationale for creating the dataset(s) clearly described?

Partly

Are the datasets clearly presented in a useable and accessible format?

Yes

Are the protocols appropriate and is the work technically sound?

Yes

Reviewer Expertise:

Genome Assembly, Genome Annotation, FAIR data

I confirm that I have read this submission and believe that I have an appropriate level of expertise to confirm that it is of an acceptable scientific standard.

# Reviewer report: Reviewer response for version 2

**DOI:** 10.21956/wellcomeopenres.25256.r108772

**Published:** 2024-11-11

**Authors:** Iván Ballester-Torres

**Affiliations:** 1 Research Institute CIBIO (Centro Iberoamericano de la Biodiversidad), Science Park, University of Alicante, Alicante, Spain

Authors have incorporated changes that answer the comments made in the anterior version. I have no further comments about the data note.

Are sufficient details of methods and materials provided to allow replication by others?

Yes

Is the rationale for creating the dataset(s) clearly described?

Yes

Are the datasets clearly presented in a useable and accessible format?

Yes

Are the protocols appropriate and is the work technically sound?

Yes

Reviewer Expertise:

Entomology, Taxonomy and Systematics in Syrphidae, specifically in the genus Cheilosia

I confirm that I have read this submission and believe that I have an appropriate level of expertise to confirm that it is of an acceptable scientific standard.

# Reviewer report: Reviewer response for version 2

**DOI:** 10.21956/wellcomeopenres.25256.r108771

**Published:** 2024-11-01

**Authors:** Arun Arumugaperumal

**Affiliations:** 1 Department of Biotechnology, Rajalakshmi Engineering College, Chennai, Tamilnadu, India

Approved. The first author could give a more complete address or create an ORCID ID and link to this article.

Are sufficient details of methods and materials provided to allow replication by others?

Yes

Is the rationale for creating the dataset(s) clearly described?

Yes

Are the datasets clearly presented in a useable and accessible format?

Yes

Are the protocols appropriate and is the work technically sound?

Yes

Reviewer Expertise:

Genomics, Bioinformatics

I confirm that I have read this submission and believe that I have an appropriate level of expertise to confirm that it is of an acceptable scientific standard.

# Reviewer report: Reviewer response for version 1

**DOI:** 10.21956/wellcomeopenres.21679.r84167

**Published:** 2024-09-27

**Authors:** Arun Sethuraman, Priyanshi Shah

**Affiliations:** 1 Department of Biology, San Diego State University, San Diego, California, USA; 2 Biology, San Diego State University College of Sciences (Ringgold ID: 115104), San Diego, California, USA

The authors clearly mention the “why” behind this genome assembly. European hawkweeds are one of the troublesome weeds that have become invasive outside of their native range. Thus, biological control of such invasive species is crucial, especially when mechanical control methods are difficult and ineffective. The introduction of natural enemies or predators is one of the ways in which we can recreate/ensure such balance.

 Using the 3C criterion (contiguity, completeness, and correctness) for genome assembly evaluation, the authors present a highly contiguous assembly with a 97.1% score on the BUSCO v5.3.2 completeness scale, with minimal duplicated sequences (0.7%). They have also assembled the X-sex chromosome and mitochondrial genome. The sections and paragraphs are coherent, and figures and tables are appropriately labeled and aligned with the text. We found the manuscript itself to be very informative, with sufficient detail. The genome will be a great resource to the Diptera research community and will surely aid in understanding this hoverfly and its ecology.

**We, however, had just a few suggestions and comments that would help improve this resource:**

 1. While the assembly, annotation, and quality assessment methods are well described and the metadata link is provided, it would greatly help the community if the authors were to share their code/scripts/pipeline via a GitHub/Zenodo page. This would also ensure the reproducibility of the methods. 

 2. Since it was mentioned that *Cheilosia pagana *and *Cheilosia vulpina* assemblies were used as comparators to identify the X chromosome for *C. urbana*, we do agree with Iván Ballester-Torres’s comment on adding taxonomic complexity of Cheilosia due to the morphological variability and the resemblance among species into the manuscript.

Are sufficient details of methods and materials provided to allow replication by others?

Partly

Is the rationale for creating the dataset(s) clearly described?

Yes

Are the datasets clearly presented in a useable and accessible format?

Partly

Are the protocols appropriate and is the work technically sound?

Yes

Reviewer Expertise:

Genomics, population genetics, computational biology, biological control.

We confirm that we have read this submission and believe that we have an appropriate level of expertise to confirm that it is of an acceptable scientific standard.

# Reviewer report: Reviewer response for version 1

**DOI:** 10.21956/wellcomeopenres.21679.r87185

**Published:** 2024-07-26

**Authors:** Arun Arumugaperumal

**Affiliations:** 1 Department of Biotechnology, Rajalakshmi Engineering College, Chennai, Tamilnadu, India

This is the first report of the whole genome of hawkweed *Cheilosia urbana*. The genome size reported was 546.9 Mbp. The assembly consisted of 5 pseudochromosomes including one X chromosome. A closely related species *C. vernalis *had its genome sequenced and was reported to be 441.6 Mbp with 6 pseudochromosomes including one X chromosome. Although the technologies used for sequencing were similar except for the fold coverage, there was a huge difference in the genome assembly in terms of size and number of chromosomes. This makes way to comparison studies between the species using the genomic data. The sequencing data is very useful in getting insights in this angle.

 The assembly consisted of 152 contigs with N50 value of 172 Mb. Long read sequencing technology was used and the Pacific Biosciences SEQUEL II machine was used to sequence the samples with 32X coverage. BUSCO analysis indicates that there is a completeness of 97 percentage. Taken all together this data provides a valuable source for further research about the insect.

 The hawkweed plant is an alien invasive species and this insect feeds on the plant in its larva stage and controls the spread. I think the genome data reported herewith will help to study the relationship between the plant and the animal in greater depths. However the genome of the plant is yet to be sequenced. 

 The accession numbers presented here are working fine and the methods used are technically sound. Hence the data article can be accepted for indexing.

Are sufficient details of methods and materials provided to allow replication by others?

Yes

Is the rationale for creating the dataset(s) clearly described?

Yes

Are the datasets clearly presented in a useable and accessible format?

Yes

Are the protocols appropriate and is the work technically sound?

Yes

Reviewer Expertise:

Genomics, Bioinformatics

I confirm that I have read this submission and believe that I have an appropriate level of expertise to confirm that it is of an acceptable scientific standard.

## References

[1] : The genome sequence of the yarrow Cheilosia, Cheilosia vernalis (Fallén, 1817). *Wellcome Open Research* .2024;9: 10.12688/wellcomeopenres.21219.1 10.12688/wellcomeopenres.21219.1

# Reviewer report: Reviewer response for version 1

**DOI:** 10.21956/wellcomeopenres.21679.r87194

**Published:** 2024-07-23

**Authors:** Thomas Brown

**Affiliations:** 1 Leibniz IZW (Berlin) & ERGA/BGP, Leibniz, Germany

Falk et al. present the genome assembly of the hawkweed Cheilosia based on PacBio HiFi and Illumina HiC reads. The genome itself is of high quality and will certainly aid researchers in their genetic explorations of this phylum, however I feel there are a number of issues that need to be addressed in this manuscript:

**Introduction:**

 It is made clear that this species of global interest given its role as an invasive species threatening the environment in some countries, however it is not made clear how having a reference genome will assist in combating this issue. A more thorough explanation of the uses of genetic resources in such activities is needed here.

**Assembly QC and methods:**

 I was astonished to see a kmer completeness of 100% and was intrigued enough to download the HiFi reads and assemblies myself to see what such a genome looks like. I was not able to recreate the 100% kmer completeness and indeed this value was much lower when considering each assembly separately. (meryl v1.3, merqury v1.3)

 merqury.out.qv:

 GCA_946477585.1_idCheUrba1.1_genomic.fna 5256 546914572 65.0862 3.1001e-07

 GCA_946477595.1_idCheUrba1.1_alternate_haplotype_genomic.fna 4047 536381505 66.137 2.43388e-07

 Both 9303 1083296077 65.5748 2.77023e-07

 completeness.stats

 CA_946477585.1_idCheUrba1.1_genomic.fna all 365075436 544766107 67.0151

 GCA_946477595.1_idCheUrba1.1_alternate_haplotype_genomic.fna all 361091971 544766107 66.2839

 both all 538481402 544766107 98.8463

 Could the authors please clarify the commands and datasets used in order to calculate this value? Does the value reported correspond to both assembled haplotypes? This is inconsistent with the rest of the table and should be made clearer. Was a filtering performed on the reads to obtain this high completeness value? I find the methods section quite opaque in these regards and would like to see more clarity throughout.

Are sufficient details of methods and materials provided to allow replication by others?

Partly

Is the rationale for creating the dataset(s) clearly described?

Partly

Are the datasets clearly presented in a useable and accessible format?

Yes

Are the protocols appropriate and is the work technically sound?

Yes

Reviewer Expertise:

Genome Assembly, Genome Annotation, FAIR data

I confirm that I have read this submission and believe that I have an appropriate level of expertise to confirm that it is of an acceptable scientific standard, however I have significant reservations, as outlined above.

# Response

**Published:** 2024-08-05

**Authors:** Tree of Life Team Sanger

**Affiliations:** Wellcome Trust Sanger Institute, UK

Thank you for your review. We have now expanded on the methods used for genome evaluation under the heading “Evaluation of genome assembly”. We’ve rerun the pipeline and the values are the same as reported in the data note: 100% completeness and QV 65.1.   Assembly        Region  Found   Total   % Covered GCA_946477585.1 all     544760851       544766107       100.00   Assembly        No Support      Total   Error % QV GCA_946477585.1 5256    546914572       0.0000  65.1

 Here is some further information about the methods:

- The *k*-mer database is made of the PacBio reads from ERR9981095 and *k*=31. This is constructed directly after the raw data is received, and reads with adapter sequences are filtered out.
- The value corresponds to the primary assembly only.
- We use FastK and MerquryFK, not meryl and merqury.
- The MerquryFK  command is at https://github.com/sanger-tol/genomenote/blob/1.0.0/modules/nf-core/merquryfk/merquryfk/main.nf#L44-L50

Please note that we have supplied the full code used for the evaluation steps in the GitHub links given in Table 3.

# Reviewer report: Reviewer response for version 1

**DOI:** 10.21956/wellcomeopenres.21679.r63752

**Published:** 2023-09-04

**Authors:** Iván Ballester-Torres

**Affiliations:** 1 Research Institute CIBIO (Centro Iberoamericano de la Biodiversidad), Science Park, University of Alicante, Alicante, Spain

The article is clear and well-conducted. 

 I cannot make any comments about the methods of genome sequencing, as I have been working in barcoding and phylogenetic trees.

 Regarding the "Background" section, there are several aspects that the authors should revise:

 - The first time a scientific name of a plant is mentioned must be followed by the author name of the taxon in the correct form. This should be added for the following species in the paper: *Acer pseudoplatanus, Anemone nemorosa, Prunus spinosa* and *Hieracium pilosella, *as well as all the mentioned plant genera. 

 - The authors could mention the assessment of *C. urbana *in the IUCN Red List of Hoverflies and its category (citation 1), in relation to the present study.

 - It might be interesting to add a few words about the taxonomic complexity of *Cheilosia* due to the morphological variability and the great resemblance between species, and how these new molecular tools can help to unravel the taxonomic problems ocurring in some species groups of *Cheilosia* and other hoverfly genera. As a result of this, the authors could mention the need of conduct a European revision of the *Cheilosia mutabilis *species group (where *C. urbana* belongs).

 Lastly, I highly recommend that the authors take into account the Atlas of the Hoverflies of Great Britain (Diptera, Syrphidae) (Citation 2), where *C. urbana *is mentioned, with some additional data that could be of interest for the authors.

Are sufficient details of methods and materials provided to allow replication by others?

Yes

Is the rationale for creating the dataset(s) clearly described?

Yes

Are the datasets clearly presented in a useable and accessible format?

Yes

Are the protocols appropriate and is the work technically sound?

Yes

Reviewer Expertise:

Entomology, Taxonomy and Systematics in Syrphidae, specifically in the genus Cheilosia

I confirm that I have read this submission and believe that I have an appropriate level of expertise to confirm that it is of an acceptable scientific standard, however I have significant reservations, as outlined above.

## References

[1] : Cheilosia urbana (Europe assessment). The IUCN Red List of Threatened Species 2021: e.T149163558A149163561. 2021; 10.2305/IUCN.UK.2021-3.RLTS.T149163558A149163561.en 10.2305/IUCN.UK.2021-3.RLTS.T149163558A149163561.en

[2] : Atlas of the Hoverflies of Great Britain (Diptera, Syrphidae). *Wallingford, Biological Records Centre* .2011;
